# Acute Respiratory Infections in Children: A Narrative Perspective on Integrated Primary–Hospital Care in Italy

**DOI:** 10.3390/children13091203

**Published:** 2026-09-06

**Authors:** Cristiana Indolfi, Angela Klain, Giulio Dinardo, Carolina Grella, Salvatore Cascone, Michele Miraglia del Giudice

**Affiliations:** Department of Woman, Child and of General and Specialized Surgery, University of Campania Luigi Vanvitelli, 80138 Naples, Italy; cristianaind@hotmail.com (C.I.); angela.klain@unicampania.it (A.K.); carolina.grella@studenti.unicampania.it (C.G.); salvatore.cascone@studenti.unicampania.it (S.C.); michele.miragliadelgiudice@unicampania.it (M.M.d.G.)

**Keywords:** acute respiratory infections, children, primary care, hospital care, continuity of care, respiratory syncytial virus, Nirsevimab, RespiVirNet, antimicrobial stewardship

## Abstract

**Highlights:**

**What are the main findings?**

- Pediatric acute respiratory infections remain a major burden on both primary and hospital care, with marked variability in referral, hospitalization, and antibiotic prescribing practices.
- An integrated primary–hospital approach based on shared referral criteria, structured information transfer, antimicrobial stewardship, prevention, and common quality indicators may improve coordination of care.

**What are the implications of the main findings?**

- Shared, disease-specific pathways may support appropriate referral, continuity of care, and more judicious antibiotic use across primary and hospital pediatric settings.
- The proposed framework should be prospectively evaluated using standardized clinical and quality-of-care indicators before its impact on outcomes and healthcare utilization can be established.

**Abstract:**

Acute respiratory infections (ARIs) remain one of the leading causes of pediatric consultations, emergency department visits, and hospital admissions worldwide, imposing a substantial clinical, organizational, and economic burden on healthcare systems. Although most ARIs are self-limiting viral illnesses, their high incidence during childhood continues to challenge both primary care pediatricians and hospital services. In recent years, the epidemiology and prevention of pediatric respiratory infections have evolved considerably following the COVID-19 pandemic and the introduction of nirsevimab immunization against RSV, while RespiVirNet has strengthened national respiratory-virus surveillance. These developments reinforce the need for integrated models of care. This narrative perspective discusses the current epidemiology and burden of pediatric ARIs, predominantly within the Italian healthcare setting, and examines the complementary roles of family and hospital pediatricians in clinical assessment, risk stratification, appropriate referral, and continuity of care. Particular attention is paid to antimicrobial stewardship and strategies to reduce inappropriate antibiotic prescribing. Preventive strategies, including RSV prevention and influenza, pneumococcal, and pertussis vaccination, are also considered as integral components of coordinated pediatric respiratory care. Finally, we discuss how surveillance, preventive interventions, shared referral criteria, structured information transfer, and measurable quality indicators could support more coordinated primary–hospital care and provide a framework for prospective evaluation.

## 1. Literature Approach

This narrative perspective was informed by a structured search of PubMed/MEDLINE and by consultation of official documents from the World Health Organization (WHO), European Centre for Disease Prevention and Control (ECDC), Italian National Institute of Health (ISS), Italian Ministry of Health, and National Institute for Health and Care Excellence (NICE). The literature search focused on studies published from January 2015 to August 2026, with earlier landmark studies included when directly relevant. Search terms combined “child” or “pediatric” with “acute respiratory infection”, “bronchiolitis”, “pneumonia”, “respiratory syncytial virus”, “nirsevimab”, “antimicrobial stewardship”, “primary care”, “emergency department”, “integrated care”, “point-of-care testing”, and “lung ultrasound”. English- and Italian-language sources were considered. References were selected according to their relevance to pediatric ARI epidemiology, prevention, diagnosis, antimicrobial stewardship, and primary–hospital care integration, prioritizing clinical guidelines, surveillance reports, systematic reviews, and recent primary studies. The literature search was last updated on 29 August 2026.

## 2. Acute Respiratory Infections in Childhood: Epidemiology and Disease Burden

Acute respiratory infections (ARIs) remain one of the leading causes of pediatric healthcare utilization worldwide and represent a major public health challenge, particularly during the first years of life. They account for a substantial proportion of primary care consultations, emergency department (ED) visits, hospital admissions, and healthcare expenditure, placing a considerable burden on children, families, and healthcare systems. Although most ARIs are self-limiting viral illnesses, their high incidence translates into a significant clinical, organizational, and economic impact, especially during the winter season when multiple respiratory viruses circulate simultaneously [1,2,3,4]. Within the broader spectrum of pediatric ARIs, lower respiratory tract infections (LRTIs) account for a disproportionate share of severe disease. According to the Global Burden of Disease 2023 study, LRTIs continue to rank among the leading causes of morbidity and mortality in children younger than five years, despite the remarkable progress achieved through vaccination programs and improvements in pediatric healthcare [1]. Infants are particularly vulnerable to severe lower respiratory tract disease because of their anatomical and immunological immaturity, smaller airway caliber, and limited respiratory reserve, factors that increase the risk of hospitalization and respiratory failure [1,2]. In addition to their direct clinical consequences, ARIs generate a substantial indirect burden through parental work absenteeism, increased healthcare utilization, inappropriate antibiotic prescriptions, and seasonal overcrowding of emergency departments and pediatric wards [2,3,4]. Italian surveillance has documented the circulation of influenza viruses, RSV, rhinoviruses, human metapneumovirus, SARS-CoV-2, and other respiratory viruses [5]. To monitor these epidemiological trends, Italy operates RespiVirNet, the national epidemiological and virological surveillance system coordinated by the Italian National Institute of Health (Istituto Superiore di Sanità). The network integrates data collected by family pediatricians, general practitioners, and reference laboratories, providing continuous surveillance of respiratory virus circulation and supporting public health planning. During the 2024–2025 season, RespiVirNet epidemiological surveillance was based on the influenza-like illness (ILI) case definition. More than 16 million ILI cases were estimated over the season, representing the highest absolute number recorded since the start of surveillance. The epidemic curve reached a peak incidence of 17.4 cases per 1000 persons under surveillance in week 4 of 2025, which was lower than the peak observed during the previous season (18.4 per 1000). Children aged 0–4 years consistently showed the highest incidence. Virological surveillance documented the co-circulation of influenza viruses, RSV, rhinoviruses, human metapneumovirus, and other respiratory viruses [5,6]. From the 2025–2026 season, RespiVirNet adopted the acute respiratory infection (ARI) case definition to align Italian surveillance with ECDC standards. The system integrates case reporting by sentinel general practitioners and family pediatricians with virological data collected across community and hospital settings, providing a broader framework for monitoring respiratory infections at the national level [6]. Because surveillance in 2024–2025 was still based on the ILI definition, comparisons of incidence across the two seasons should be interpreted with caution. Italian real-world data further emphasize the burden of RSV in early childhood. In a retrospective observational study conducted at the University Hospital of Foggia, 1005 laboratory-confirmed RSV cases were identified between 2011 and 2023. However, the clinical and economic analyses were based on the 832 RSV cases from 2011–2020 that could be matched with the corresponding hospital discharge records [4]. Within this 2011–2020 cohort, 75.2% of hospitalized children were younger than one year, and 49.6% were aged 0–2 months. Bronchiolitis was the most frequent admission diagnosis (63.3%), and 25% of children were classified as having very severe RSV disease. Among children younger than one year, RSV accounted for 48.9% of hospitalizations for acute respiratory infections. The mean cost of an RSV-associated hospitalization was €3036 (95% CI: €2643–€3428), increasing to €4225 (95% CI: €3474–€4977) among infants aged 0–2 months [4].

## 3. Nirsevimab Immunization and the Changing Landscape of Pediatric Respiratory Infections

The introduction of nirsevimab immunization during the 2024–2025 RSV season represented an important expansion of RSV prevention in Italian infants. Nirsevimab, a long-acting monoclonal antibody providing passive immunization throughout the RSV season, has demonstrated high effectiveness in reducing medically attended RSV infections across different healthcare settings, with a particularly relevant impact in infants during their first months of life, when the risk of severe bronchiolitis and hospitalization is highest [7,8]. However, implementation was regionally coordinated rather than uniform across the country, with differences in campaign timing and eligibility criteria [9]. In a multicenter Italian retrospective comparative study involving nine hospitals, bronchiolitis admissions decreased by 48% during the 2024–2025 season compared with the previous season (438 vs. 832 admissions) [10]. Consistently, a nationwide Italian study found that nirsevimab immunization was associated with lower odds of RSV-related hospitalization (aOR 0.259; 95% CI 0.157–0.427), corresponding to an estimated effectiveness of 74.1% (95% CI 57.3–84.3) [11]. Following the introduction of nirsevimab immunization, Italian multicenter studies have also reported a relative change in the viral profile of infants hospitalized with bronchiolitis or lower respiratory tract infections, with RSV becoming less dominant and a greater relative representation of non-RSV pathogens, particularly rhinovirus/enterovirus and human metapneumovirus [10,11]. These early observational findings may suggest a shift in the relative distribution of respiratory pathogens among hospitalized infants; however, they should not be interpreted as evidence of pathogen replacement or of an increased population incidence of non-RSV infections. Importantly, an exploratory post hoc analysis of the randomized phase 3 MELODY trial found no overall increase in non-RSV medically attended LRTIs among nirsevimab recipients and no evidence of replacement of RSV by other respiratory viruses during the study period [12]. Nevertheless, the low incidence of some pathogens, including hMPV, limited definitive conclusions, supporting the need for continued real-world surveillance and larger prospective population-based studies across different countries and healthcare settings. Recent international surveillance data have also highlighted marked between-country heterogeneity in rhinovirus activity and epidemic duration [13]. These observations reinforce the need for an integrated model of pediatric respiratory care based on close collaboration between primary care pediatricians and hospital services. Beyond preventing severe RSV disease at the individual level, nirsevimab immunization may represent an important component of broader RSV prevention strategies and may contribute to reducing seasonal healthcare demand [7,10]. Observed reductions in RSV-related hospitalizations may therefore facilitate more appropriate allocation of pediatric healthcare resources during seasonal epidemics, although their broader impact on integrated care pathways requires further evaluation. Despite these encouraging findings, several aspects should be considered when interpreting the population-level impact of nirsevimab. Post-implementation data should also be interpreted in light of heterogeneous regional implementation. In two Italian multicenter cohorts of hospitalized infants, 23% and 40.4%, respectively, had previously received nirsevimab [10,11]; these proportions should not be interpreted as estimates of population-level coverage. Moreover, the cost-effectiveness and sustainability of large-scale immunization programs depend on acquisition costs, healthcare organization, population coverage, and local RSV burden [9,14]. Differences in regional implementation and access may also influence their real-world impact, highlighting the importance of continued surveillance and evaluation of both effectiveness and equity [9].

## 4. Bridging Primary and Hospital Care: The Central Role of the Primary Care Pediatrician

Primary care pediatricians are central to deciding whether a child with an acute respiratory infection can be safely managed in the community or requires hospital assessment. This decision should integrate clinical severity, age, underlying conditions, feeding and hydration status, caregiver ability to monitor the child, and the availability of appropriate follow-up [15,16,17]. Prevention is an integral part of this role. Beyond RSV prevention with nirsevimab, age-appropriate immunization against influenza, pneumococcal disease, and pertussis should be routinely reviewed. In Italy, seasonal influenza vaccination is actively recommended and offered free of charge to healthy children aged 6 months to 6 years inclusive and to older children at increased risk of influenza-related complications [18]. Pneumococcal and pertussis vaccinations are included in the national childhood immunization schedule [18], while pertussis vaccination is also recommended during each pregnancy, ideally around the 28th week of gestation [19]. Family pediatricians can identify missed vaccination opportunities, counsel families, and, according to regional organization, participate directly in vaccine delivery. Vaccination coverage may therefore represent a measurable quality indicator within integrated care pathways. Preventive counseling should also reinforce hand and respiratory hygiene and avoidance of school or day-care attendance during symptomatic acute respiratory infections [20,21]. Most uncomplicated respiratory infections can be managed with supportive care, caregiver education, safety-netting, and clinical reassessment when needed [15,17]. Referral thresholds should, however, reflect the specific respiratory syndrome. In bronchiolitis, immediate hospital referral is recommended for apnea, severe respiratory distress, central cyanosis, or a seriously unwell appearance, while referral should also be considered in the presence of respiratory rate > 60 breaths/min, inadequate oral intake or dehydration, or persistent oxygen saturation < 92% in room air [17]. Younger age, particularly <3 months, prematurity, chronic lung disease, hemodynamically significant congenital heart disease, neuromuscular disorders, and immunodeficiency further increase the risk of severe disease [17]. In community-acquired pneumonia, features such as hypoxemia, marked respiratory distress, severe chest indrawing, inability to drink, lethargy, or reduced consciousness should prompt consideration of hospital referral or specialist pediatric assessment [16]. Italian data nevertheless show substantial variability in hospital use. In the multicenter CHOICE study, which included 3145 children with ARI and no emergency or priority signs across 11 pediatric emergency departments, hospitalization rates ranged from 2.3% to 30.6%. After adjustment, hospitalization rates were higher in facilities in Southern Italy (+24.7%; 95% CI 17.8–31.5) and lower in university centers (−13.1%; 95% CI −18.6 to −7.6) [3]. Organizational heterogeneity has also been reported by a national SIMEUP survey, which identified differences among pediatric emergency settings in infection-control resources, availability of information, and dedicated pediatric pathways [22]. Together, these findings suggest that clinical disposition is influenced not only by patient characteristics but also by local practice and healthcare organization. Reducing unwarranted variability therefore requires closer coordination between primary and hospital care. The framework proposed here is based on three practical components: (i) shared disease-specific referral criteria; (ii) standardized bidirectional transfer of essential clinical information at referral and discharge; and (iii) common quality indicators across care settings. Potential measures include hospitalization and emergency department revisit rates, guideline-concordant antibiotic prescribing, completion of follow-up, timely transmission of discharge information, and vaccination coverage (Table 1). This framework should be considered a proposal for prospective evaluation rather than an established model of care, and its impact should be assessed using predefined clinical and organizational outcomes [3].

## 5. Antimicrobial Stewardship in Pediatric Acute Respiratory Infections: From Diagnostic Uncertainty to Appropriate Prescribing

Respiratory infections account for a substantial proportion of antibiotic prescriptions in childhood, although antibiotics are not indicated for uncomplicated viral ARIs [23]. Unnecessary or inappropriate antibiotic exposure contributes to antimicrobial resistance (AMR), making appropriate prescribing a priority across primary and hospital care [23,24]. Distinguishing viral from bacterial infections can be challenging because of overlapping clinical presentations, while commonly used investigations including white blood cell count, C-reactive protein, procalcitonin, and chest radiography have limited discriminatory value when considered in isolation [25,26]. Diagnostic uncertainty and parental expectations may therefore contribute to inappropriate prescribing, highlighting the importance of careful clinical assessment, effective communication, safety-netting, and, when appropriate, watchful waiting or delayed prescribing [23]. Substantial variation in prescribing practice remains evident. In the CHOICE cohort of children with ARI and no emergency or priority signs, antibiotic prescription rates ranged from 22.6% to 80.0%; facilities in Southern Italy had higher prescribing rates (+33.1%; 95% CI 20.4–45.9) [3]. Temporal changes in prescribing have also been reported: in a single-center Italian retrospective cohort of 4882 febrile ARI episodes, Pierantoni et al. reported a reduction in antibiotic prescribing at discharge from 56.7% in 2016–2017 to 40.6% in 2023–2024 (*p* < 0.001), together with a shift towards narrower-spectrum agents [27]. Given the before–after design, however, this change should not be interpreted causally. Together, these findings highlight substantial variability in antibiotic prescribing across settings and over time, supporting the value of shared clinical pathways and prescribing reviews across primary and hospital care.

Antibiotic indications should also be considered within specific clinical syndromes. Antibiotics are not recommended for uncomplicated bronchiolitis, whereas suspected bacterial community-acquired pneumonia (CAP) and microbiologically confirmed streptococcal pharyngitis generally warrant antimicrobial treatment; selected cases of acute otitis media may also require antibiotics [16,17,28,29,30,31]. For uncomplicated mild-to-moderate CAP and confirmed streptococcal pharyngitis, recent Italian intersociety recommendations identify amoxicillin as the preferred first-line agent [29,30]. A key stewardship issue, however, is the unnecessary use of broader-spectrum alternatives. Italian primary-care data show substantial use of amoxicillin–clavulanate and cephalosporins in children [32]. The same pattern has been documented in acute pharyngitis, where amoxicillin–clavulanate was reported in approximately 25% of Italian pediatric emergency units and third-generation cephalosporins in approximately 28% of primary-care cases [30]. In acute otitis media, current Italian recommendations similarly favour narrow-spectrum amoxicillin when antibiotic therapy is indicated, reserving amoxicillin–clavulanate or cephalosporins for selected circumstances [28]. Thus, appropriate prescribing involves not only avoiding antibiotics when they are unnecessary but also avoiding unwarranted escalation to broader-spectrum agents. Recent epidemiological changes add further complexity. A post-pandemic re-emergence of Mycoplasma pneumoniae has been documented across multiple European countries and other geographical regions [33]. In an Italian pediatric center, the proportion of M. pneumoniae among ARI hospitalizations increased from 3.2% in 2019 to 6.1% in 2024 [34]. Pertussis also showed a marked resurgence across Europe, with 209,674 cases reported in 29 EU/EEA countries in 2024, with infants younger than one year among the most affected age groups [35]. Recognition of specific bacterial pathogens can guide targeted therapy and avoid unnecessary empirical antibiotic use. Overall, stewardship should prioritize selective diagnostics, narrow-spectrum treatment when indicated, and shared prescribing strategies across primary and hospital care, with performance monitored through antibiotic use and guideline-concordant prescribing indicators (Table 2).

## 6. Lung Ultrasound as a Point-of-Care Tool for Early Assessment of Pediatric Respiratory Infections

Although most pediatric acute respiratory infections are self-limiting, persistent fever and cough associated with tachypnea, respiratory distress, hypoxemia, focal auscultatory findings, or clinical deterioration should raise suspicion of pneumonia or other lower respiratory tract complications. In this setting, lung ultrasound (LUS) represents a useful bedside diagnostic tool because it is rapid, repeatable, radiation-free, and increasingly available in pediatric practice. LUS can identify findings suggestive of pneumonia, including subpleural consolidations with air bronchograms, focal or asymmetric B-line patterns, pleural abnormalities, and pleural effusion, thereby supporting the early recognition of pneumonia and its complications [36,37,38]. Recent meta-analytic evidence including 30 studies and 4546 children showed a pooled sensitivity of 94.0% and specificity of 85.5% for LUS in the diagnosis of pediatric community-acquired pneumonia [39]. When adequate equipment and specific training are available, LUS may also support primary care pediatricians in assessing children with persistent or worsening respiratory symptoms and in guiding decisions regarding outpatient follow-up, further investigation, or timely hospital referral [40]. Moreover, serial LUS examinations may support the follow-up of children with pneumonia, particularly in more severe cases, by enabling radiation-free monitoring of the evolution and resolution of pulmonary abnormalities over time [41]. LUS may represent an additional tool to strengthen risk stratification, continuity of care, and the interface between primary and hospital care. Although some ultrasound patterns may differ between viral and bacterial infections, substantial overlap remains; therefore, LUS should not be considered an etiological test in isolation. Rather, ultrasound findings should be interpreted together with clinical presentation, inflammatory biomarkers, and microbiological testing when indicated. Moreover, LUS remains operator-dependent and may fail to detect lesions that do not reach the pleural surface. Accordingly, it should complement clinical assessment and be integrated with other diagnostic investigations when clinically indicated [36,37].

## 7. Limitations

This article has several limitations. First, the literature selection was narrative rather than systematic and may therefore be subject to selection bias. Second, the manuscript is predominantly focused on the Italian healthcare setting, and the proposed organizational framework may not be directly transferable to healthcare systems with different primary-care structures, referral pathways, or resources. Third, much of the available evidence on integrated primary–hospital pathways is observational or descriptive, and controlled studies demonstrating their impact on clinical outcomes and healthcare utilization remain limited. Finally, respiratory-virus epidemiology, preventive strategies, and healthcare organization are rapidly evolving, requiring continued surveillance and prospective evaluation of the proposed framework.

## 8. Conclusions

Acute respiratory infections remain a major cause of pediatric healthcare utilization, and their safe management requires early risk stratification and timely escalation of care for children at increased risk of severe disease or complications. The integrated framework proposed in this perspective is based on shared referral criteria, standardized transfer of clinical information between primary and hospital care, appropriate use of diagnostic tools, antimicrobial stewardship, and common quality indicators. Prevention through immunization and epidemiological surveillance should also form part of this coordinated approach. However, evidence that such integrated pathways improve clinical outcomes or reduce healthcare utilization remains limited. Their effectiveness should therefore be evaluated prospectively in controlled studies using predefined clinical and organizational outcomes and standardized quality-of-care indicators, including the WHO standard-based measures evaluated in the Italian CHOICE study.

## Figures and Tables

**Table 1:** Main challenges in the management of acute respiratory infections in pediatric age and potential improvement strategies along the hospital–community care pathway.

Domain	Identified Challenges	Improvement Strategies	Potential Quality Indicators *
Communication and continuity of care	Communication between primary care pediatricians and hospitals is not always structured; limited sharing of clinical information; difficulties in post-discharge follow-up [15].	Shared diagnostic and therapeutic pathways, standardized discharge summaries, and timely bidirectional communication between community and hospital settings [15,16,17,22].	Follow-up completion rate; timely transmission of discharge information
Accessibility and organization of services	Inappropriate emergency department visits during epidemic peaks; organizational heterogeneity across regions; variable availability of pediatric observation units and dedicated care pathways [3,22].	Strengthening primary care services and defining shared referral criteria for emergency department access [15,16,17,22].	Hospitalization rate; ED revisit rate
Family education and engagement	Limited knowledge of the natural course of respiratory infections; difficulty in recognizing warning signs [17,23].	Health education during clinical visits, use of safety-netting, shared informational materials, and active family involvement in home monitoring of the child [17,23].	Documented safety-netting; unplanned reconsultation rate
Epidemiological monitoring and quality of care	Variability in the collection of clinical and prescribing data; limited availability of shared indicators to assess appropriateness and quality of care [3,23].	Strengthening epidemiological surveillance (e.g., RespiVirNet), monitoring vaccination coverage and other quality indicators, conducting periodic audits, and implementing antibiotic stewardship programs [3,6,18,19,23].	Vaccination coverage; proportion of antibiotic prescriptions concordant with guideline recommendations; periodic audit completion

* Potential quality indicators are proposed by the authors for prospective evaluation of the integrated care framework and should not be interpreted as validated outcome measures.

**Table 2:** Principles of Antimicrobial Stewardship in the Management of Pediatric Acute Respiratory Infections.

Critical Issues	Recommended Approach	Objective
Diagnostic uncertainty in distinguishing viral from bacterial respiratory infections [23,25,26].	Careful clinical assessment, use of validated clinical criteria, and selective use of rapid antigen, molecular, or other microbiological assays when results may influence clinical management; avoidance of routine antibiotic prescribing in uncomplicated cases [16,23,27].	Reduce unnecessary antibiotic use [23,24].
Variability in clinical practice [3,32].	Apply shared guidelines and diagnostic–therapeutic pathways between primary care and hospital settings [23,28,29,30].	Standardize the management of children with ARIs [3,23].
Prescribing pressure and parental expectations [23].	Health education, effective communication, safety-netting, and delayed prescribing in selected cases [17,23].	Reduce unnecessary antibiotic prescribing while maintaining appropriate clinical reassessment [23].
Diagnostic support [16,23,27].	Use microbiological tests and point-of-care tests when indicated, as support—not a substitute—for clinical evaluation [16,23,27].	Support more targeted antibiotic prescribing [23,27].

## Data Availability

No new data were created or analyzed in this study. Data sharing is not applicable to this article.
